# The Impact of Inferior Vena Cava Anomalies on Deep Vein Thrombosis: A Case Report

**DOI:** 10.7759/cureus.75385

**Published:** 2024-12-09

**Authors:** Waslah M Mansha, Pejmahn Eftekharzadeh, Shahzad Ahmed

**Affiliations:** 1 Cardiology, Lower Bucks Hospital, Bristol, USA

**Keywords:** anticoagulation., azygos vein, deep vein thrombosis, inferior vena cava anomalies, venous thromboembolism

## Abstract

Inferior vena cava (IVC) anomalies are rare congenital pathologies related to variations of agenesis, hypoplasia, or atresia, predisposing patients to thromboembolic events secondary to an alteration in venous drainage with resultant stasis. This is a case report of a 27-year-old male without significant medical history presenting for a fall after playing recreational basketball with associated pain and swelling in his left lower extremity. After his symptoms progressively worsened, he came to the emergency room for an evaluation where an ultrasound (US) of the extremity showed extensive deep vein thromboses (DVT). Despite anticoagulation therapy, his pain increasingly worsened and thus catheter-directed thrombectomy was considered. A diagnostic venogram in the cardiac catheterization lab was obtained showing an occlusive left iliofemoral DVT with thrombosis of superficial veins along with an IVC anomaly. After a dedicated abdominal computed tomography (CT) venogram, there was no evidence of any IVC anomalies. After repeat cardiac catheterization, an IVC atresia was noted on the cath lab venogram with venous drainage occurring through the azygos vein and partial contributions from the hepatic veins. This case underscores the importance of detailed imaging and consideration of rare anatomical anomalies in diagnosing unexplained or recurrent thrombotic events especially in younger patients.

## Introduction

The inferior vena cava (IVC) is a large vein that brings deoxygenated blood back to the right atrium of the heart. Congenital variations in the inferior vena cava which include atresia's, hypoplasia's and total agenesis are uncommon when compared to other vascular anomalies, with the incidence being approximately 0.3% to 0.5% in the general population and 0.6% to 2% in individuals with other forms of cardiovascular anomalies [1]. The agenesis of the inferior vena cava (AIVC) manifests as a complete lack of development of the inferior vena cava. The incidence of this specific anomaly has been reported as low as 0.0005% up to 1%. Deep vein thrombosis (DVT) associated with AIVC is frequently encountered and studies have reported AIVC prevalence in DVT cases to be as high as 5% in the young adult population [2].

The IVC embryogenesis is complex and involves numerous transformations throughout its development [3]. Any alteration of this process may lead to some form of IVC deformity most commonly characterized by the incomplete or complete absence of the inferior vena cava segment. In such instances where there are no other congenital defects, these anomalies are usually of no clinical significance as venous return is facilitated through various compensatory mechanisms, mainly utilizing the azygos and hemiazygos systems. Nonetheless, with an IVC atresia, there is a funnel neck phenomenon where all venous return from the lower extremities goes through the azygos vein. The azygous system itself will not experience clotting due to persistent blood flow back to the right atrium but the vessels behind the azygos vein will have persistent stasis of blood, predisposing them to clotting [1]​.

Defects of the IVC are frequently found incidentally either during imaging or while examining for thromboembolic complications. About 5% to 6.7% of young patients aged between 20 and 40 years old who present with DVT are found to have IVC defects based on imaging studies, which is much higher than that seen in the general population. Interestingly, 62.5% of these patients had bilateral DVT as opposed to 8.6% in the total population of acute DVT patients, likely attributed to bilateral venous stasis from both extremities affected equally [4-7].​

## Case presentation

A 27-year-old male with no significant medical history presented to the emergency department with a five-day history of progressive pain and swelling in the left lower limb. The patient reported a fall, resulting in mild bruising to his left leg, with no immediate pain or swelling. Over the next few days, his discomfort and swelling worsened, extending from the ankle to the thigh. He denied any prior thrombotic events, recent long-distance travel, immobilization, surgery, or family history of thromboembolic disorders. 

On examination, the patient was afebrile with stable vital signs. His left leg was markedly edematous from the ankle to the thigh, with increased tenderness on palpation. There was no erythema or cyanosis. Pulses were palpable in both lower extremities, but significant pitting edema was noted on the left side. The patient exhibited no signs of respiratory distress or hemodynamic instability. In the emergency room, a venous Doppler ultrasound of the left lower extremity revealed extensive occlusive DVT involving the tibial, popliteal, and femoral veins, with superficial veins also affected. After an initial hypercoagulable workup was collected (Table 1), systemic unfractionated heparin was initiated.

**Table 1:** Initial hypercoagulable workup FEU = Fibrinogen equivalent units, PT = Prothrombin time, INR = International normalized ratio, aPTT = Activated partial thromboplastin time

Laboratory test	Value	Referenc
PT	11.3s	9.7-12.2s
INR	1.04s	<4s
aPTT	27.6s	24.7-31.0s
D-dimer	16.46mg/L FEU (High)	<50mg/L FEU
Fibrinogen	477mg/dL (High)	184-403 mg/dL
Protein C	124%	73 - 183%
Protein S	74%	63-104%
Factor II	95%	50-154%
Factor V	111%	70-150%

**Figure 1:**
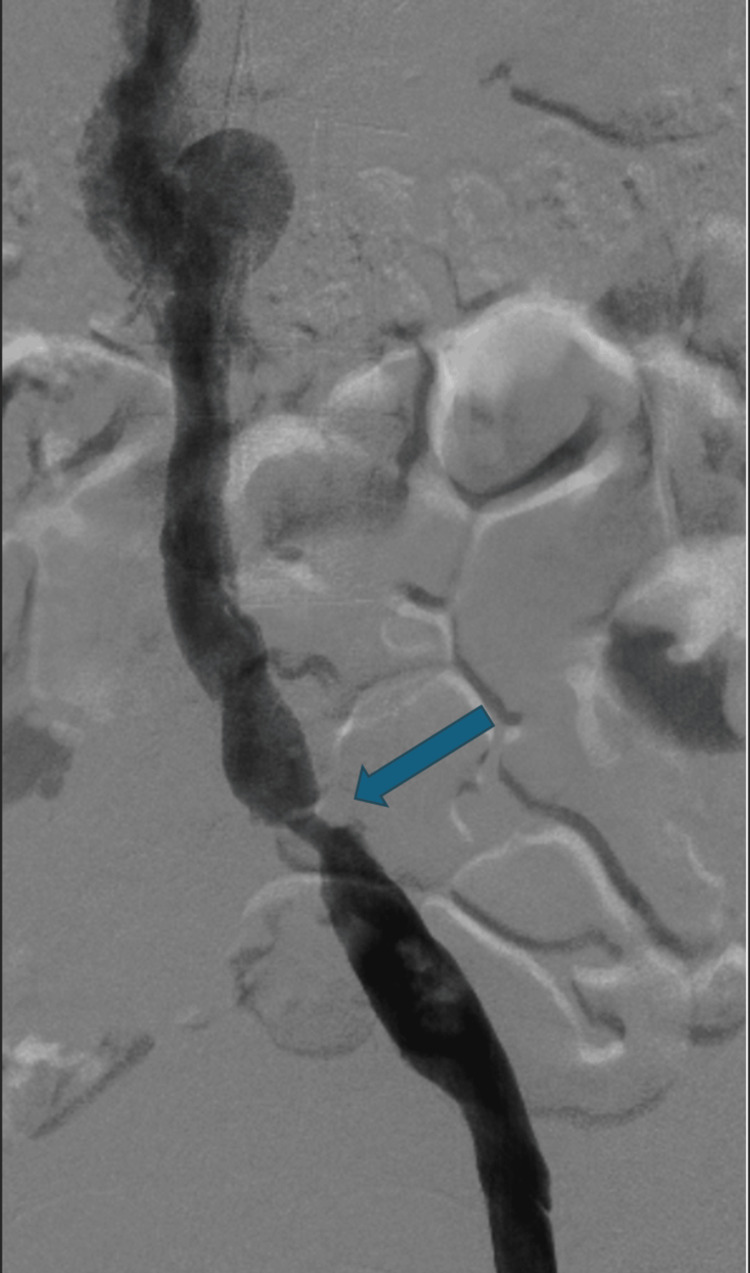
Solid arrow marks the area of stenosis in the left common iliac vein

After 24 hours of systemic anticoagulation and large clot burden, the decision was made to pursue more invasive measure for pain control which included catheter-directed thrombectomy (CDT). Thus, patient was taken to the cardiac catheterization lab for this procedure. Before undergoing CDT, a venogram was performed in the lab and not only confirmed the extensive thrombi in the venous system but also the patient was also found to have critical stenosis of the left common iliac vein (Figure 1). There was also suspicion that the IVC was aneurysmal. After the venogram, the iliac arteries were cannulated with a Cragg McNamara catheter for slow tissue plasminogen activator (tPA) infusion over the next 24 hours. After tPA infusion was initiated, a CT venogram was sought out after the procedure the same day to further investigate this poorly visualized IVC anomaly during the peripheral venogram in the catheterization lab. Unfortunately, not only was there no evidence of an aneurysmal IVC but also no reported evidence of any IVC anomaly. 

**Figure 2:**
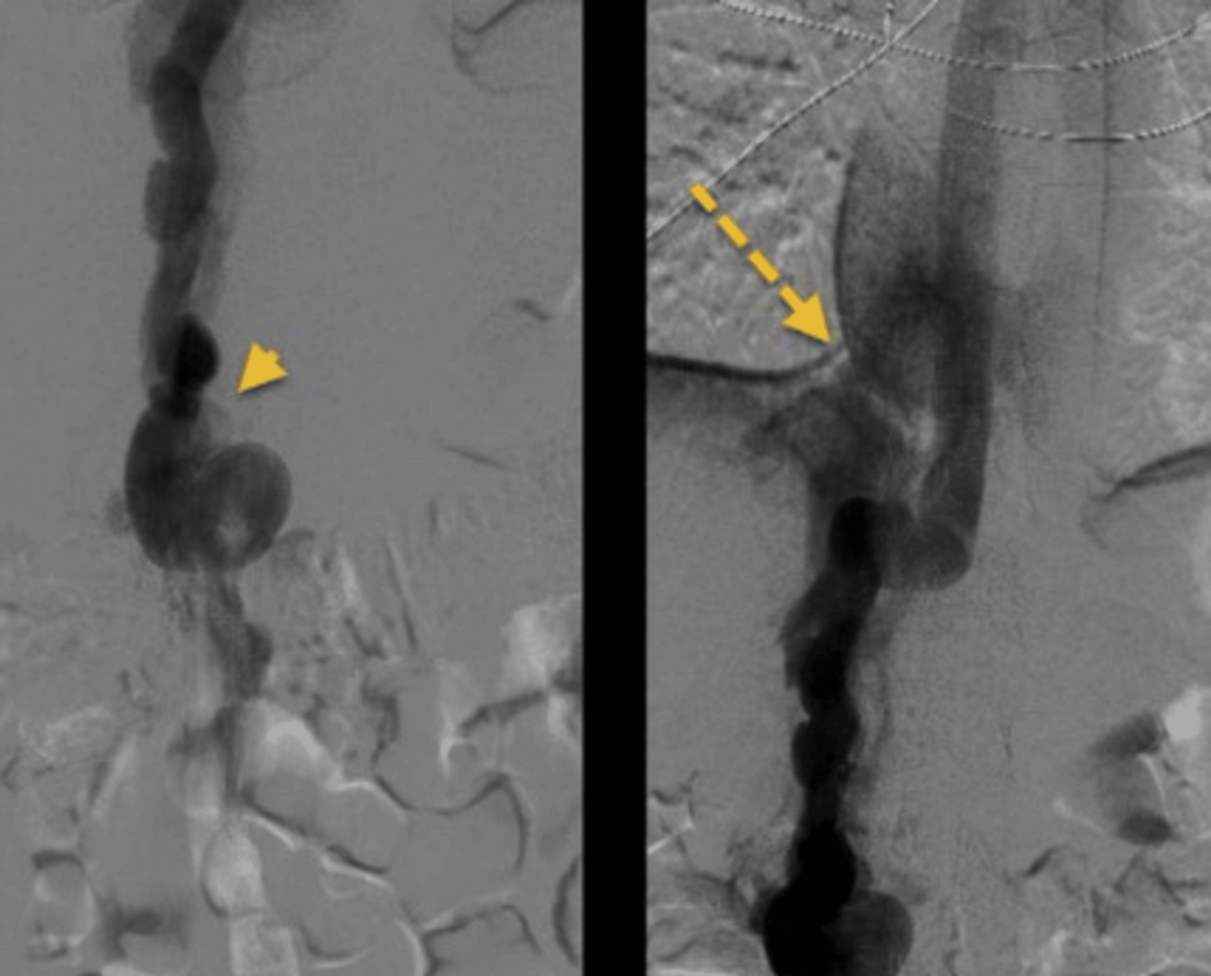
Left: Inferior vena cava (IVC) venogram showing venous drainage via the azygos vein (arrowhead) - Right: Venous drainage via the hepatic veins (broken arrow) to the superior vena cava (SVC).

The following day, the tPA catheter was removed and showed successful recannulation of the iliac and femoral veins with minimal residual thrombi noted. Next, ballon venoplasty was done on the left common iliac vein reducing the stenosis from a critical 80% to less than 30%. Following the recannulation of the majority of the venous system, repeat venogram appeared to show that the majority of venous drainage is through the azygous vein into the superior vena cava and then the right atrium (Figure 2). Due to this drainage, there was no evidence of venous return to the IVC hence an IVC atresia.

The patient was monitored overnight and was discharged the following day on rivaroxaban. The discharge instructions included follow-up for the remainder of his hypercoagulable workup as outpatient with an MRI venogram abdomen. He was also instructed to follow up with his primary care physician and the operating cardiologist for further management and assessment of long-term anticoagulation. Unfortunately, however, the patient was lost to follow-up.

## Discussion

While conventional risk factors for DVT, such as immobilization, trauma, or hypercoagulable states, may not all be present in patients presenting with a DVT, the presence of IVC anomalies should be considered in the diagnostic workup of young individuals with extensive or recurrent thrombotic events. In this case, the patient's extensive DVT was likely precipitated by baseline venous stasis from his IVC anomaly and possibly triggered after the mechanical fall as a result of vascular endothelial inflammation. The left common iliac stenosis was likely May-Turner physiology which is an anatomic variation where there is extrinsic venous compression by the arterial system against the bony structure of the axial skeleton, most commonly right common iliac artery compressing the left iliac vein against the fifth lumbar vertebra [8].

Some published works show that IVC agenesis causes marked venous stasis and as a result increases the risk of DVT because of the changes in the patterns of venous drainage. In one case report, a patient with a complete absence of IVC suffered from DVT of the iliac veins. This crucial anomaly results in elevated venous pressures within the lower limbs, leading to the formation of DVT [4]​. Conversely, another report describes a situation in which the azygous vein is the only outflow to drain blood in the absence of IVC. Still, this drainage is often associated with stasis, increasing the chances of thrombi formation, especially in the younger populations [9]​.

With a lack of risk factors and a negative hypercoagulable workup, diagnostic imaging is essential in identifying vascular anomalies such as a congenital IVC. Doppler ultrasound remains the first-line imaging modality for the diagnosis of a DVT, but it will likely fail to display anatomical anomalies given the small window of visualization. When the cause of DVT is still unclear, one may make a strong argument that it would be reasonable to escalate to broader modalities of noninvasive imaging to search for vascular anomalies for a culprit risk factor. We suggest the CT-venogram as the next best choice given that it is relatively cheaper, as compared to an MRI, and remains noninvasive. The barrier would be any patient who is unable to take contrast dye. If CT fails to show a vascular anomaly or is contraindicated, MRI would be the next best test. Our patient had an indication for invasive venography as the patient was a candidate for catheter-directed tPA and during the procedure, an invasive venogram was performed which clued the team of the IVC anomaly that was not detected on the CT venogram. No published evidence supports the use of routine invasive techniques for the diagnosis of relatively uncommon vascular anomalies at this time so the prevalence of these anomalies may be lower than actual published data. A multidisciplinary team consisting of radiologists, vascular specialists, hematologists, and in our case a cardiovascular disease specialist may all be involved in the management of acute DVT, especially in younger patients. A call for further research on the long-term outcomes of treatments like catheter-directed thrombolysis in patients with IVC anomalies may be useful in bringing awareness to the search for vascular anomalies in young patients presenting with DVT.

The importance of DVT management, treatment and risk factor modification lies in that 50% of patients with untreated symptomatic proximal DVT develop symptomatic pulmonary embolism (PE) within three months [10]. However, interestingly enough, one study demonstrated that the rate of PE occurs less frequently than expected in patients with IVC anomalies. The rate of PE is 9.7% in patients with IVC anomalies presenting with a DVT, compared to 60-80% in the general population with DVT [9]. It is hypothesized that the relatively smaller diameter of the azygos vein almost acts as a "filter" for large emboli dislodged from the downstream DVT [4]. Further research may be needed to strengthen this claim.

The literature suggests that patients should receive lifelong anticoagulation to prevent the re-occlusion of the IVC. There is no evidence to support routine surveillance imaging, but this may be a topic for further research in the future. Lifelong anticoagulation will also help remove any residual thrombi in patients who undergo catheter-directed thrombectomy as well. In one report published in Cureus, it is pointed out that although initial course of treatment differentiates in some cases, the need for long-term anticoagulation therapy is effective in lowering the rates of DVT recurrence in such patients. Another alternative for patients who are unable to tolerate lifelong anticoagulation therapy is potential surgical and endovascular repair of the anomaly. A systemic review on IVC atresia pointed out that 18.3% of published cases underwent surgical and endovascular intervention in the management of DVT provoked by IVC atresia. However, it is unclear what the rate of recurrence for DVT was for these patients [11]. Based on overwhelming case reports, the decision to offer lifelong anticoagulation is reasonable as these congenital anomalies are unprovoked unless there is surgical intervention to fix the congenital anomaly. After the discovery of such anomaly, there may be consideration for genetic testing in immediate family members to rule in or out the presence of such anomaly but further research on this topic is needed.

## Conclusions

Anomalies in the development of inferior caval veins, IVC, for instance, agenesis and hypoplasia, should always be explored in the younger population who present with unexplained or recurrent deep vein thromboses DVT. Thorough imaging evaluations are essential for the early detection of such structural anomalies to allow for proper intervention. These patients will likely need lifelong anticoagulation as this is a congenital anomaly (unprovoked) that will predispose patients to chronic venous stasis and therefore recurrent DVT's.

This case illustrates the need to explore anatomical causes such as IVC anomalies among other factors in the assessment of patients with abrupt onset extensive limb swelling and thrombus formation. It is common practice to carry out a hypercoagulable workup and age-appropriate cancer screening. If labs are unrevealing for a hematological disorder, further investigation is reasonable with noninvasive imaging to identify vascular anomalies as the culprit. Preference is always given toward noninvasive imaging unless patients have other indications for invasive venography, as did this patient who underwent catheter-directed thrombectomy which revealed such vascular anomaly.
